# Novel *GUCY2D* Gene Mutations in Japanese Male Twins with Leber Congenital Amaurosis

**DOI:** 10.1155/2015/693468

**Published:** 2015-05-13

**Authors:** Katsuhiro Hosono, Yuko Harada, Kentaro Kurata, Akiko Hikoya, Miho Sato, Shinsei Minoshima, Yoshihiro Hotta

**Affiliations:** ^1^Department of Ophthalmology, Hamamatsu University School of Medicine, 1-20-1 Handayama, Hamamatsu-shi, Higashi-ku, Shizuoka 431-3192, Japan; ^2^Department of Photomedical Genomics, Basic Medical Photonics Laboratory, Medical Photonics Research Center, Hamamatsu University School of Medicine, 1-20-1 Handayama, Hamamatsu-shi, Higashi-ku, Shizuoka 431-3192, Japan

## Abstract

*Purpose*. Leber congenital amaurosis (LCA), a genetically and clinically heterogeneous disease, is the earliest onset retinitis pigmentosa (RP) and is the most severe of hereditary retinal dystrophies. This study was conducted to investigate genetic and clinical features of LCA in a set of Japanese male twins with LCA. *Methods*. To identify causative mutations, 74 genes known to cause RP or LCA were examined by targeted-next generation sequencing (NGS). Targeted-NGS was performed using a custom designed Agilent HaloPlex target enrichment kit with Illumina Miseq sequencer. Identified potential pathogenic mutations were confirmed using Sanger sequencing. Clinical analyses were based on ophthalmic examination, fundus photography, and electroretinography (ERG). *Results*. Compound heterozygous *GUCY2D* mutations of novel splicing mutation c.2113+2_2113+3insT and novel missense mutation p.L905P were detected in both twins. Their father and mother were heterozygous for c.2113+2_2113+3insT and p.L905P, respectively. The twins had phenotypic features similar to those previously reported in patients with *GUCY2D* mutations. This included early childhood onset of visual loss, nystagmus, unrecordable ERG, photophobia, and hyperopia. *Conclusions*. To the best of our knowledge, this is the first report of genetic and clinical features of Japanese LCA twins with *GUCY2D* mutation, which were detected using targeted-NGS.

## 1. Introduction

Leber congenital amaurosis (LCA, Mendelian Inheritance in Man [MIM] 204000) is a form of retinitis pigmentosa (RP) that has the earliest onset and is the most severe form of the hereditary retinal dystrophies. The estimated prevalence of LCA is 3 per 100,000 newborn babies, but LCA accounts for at least 5% of all inherited retinal dystrophies and approximately 20% of children attending schools for the blind [1, 2].

Leber congenital amaurosis is a genetically and clinically heterogeneous disease. Clinical features of LCA include blindness or severe visual impairment at birth or within the first month of life, nystagmus, very poor or absent ocular pursuit, Franceschetti's oculodigital sign, and a severely reduced or absent electroretinogram (ERG). The fundus appearance in LCA patients varies and can appear relatively normal or have obvious pigmentary changes similar to those seen in RP patients. Other signs and symptoms which may or may not be present include photophobia, night blindness, hyperopia, macular/peripheral retinal abnormalities, keratoconus, and cataract [3–5].

A total of 21 causative genes for LCA have been found (https://sph.uth.edu/retnet/; accessed 20th January 2015). Mutations in these genes account for approximately 70% of all cases of LCA [5], with the frequency of mutation carriers varying between different populations and ethnicities [3, 6–12]. The disorder is usually inherited as an autosomal recessive (ar) trait, with an autosomal dominant inheritance pattern rarely occurring [5]. Recent clinical trials with* RPE65* replacement therapy have provided hope for LCA patients and their families. This study showed this form of gene therapy to be safe and effective in improving vision in patients with* RPE65*-associated LCA [13–16]. Gene therapy studies on animal models with other LCA-associated gene mutations are also ongoing [17–19] and will hopefully provide further effective LCA treatment strategies. Even though clinical trials are in progress, it is difficult to identify LCA patients who are eligible for gene specific treatment. Because gene therapy is mostly gene specific, an accurate molecular diagnosis of LCA patients is essential for properly identifying those patients who may benefit from upcoming novel therapies.

An accurate LCA gene analysis requires the screening of hundreds of exons in more than 20 genes. Because of the genetic heterogeneity, large-scale LCA mutation screening requires enormous effort. Currently, the most widely used methods for the molecular diagnosis of LCA are Sanger sequencing and arrayed primer extension (APEX) chips (Asper Ophthalmics, Tartu, Estonia). The Sanger sequencing technique can accurately determine sequences, but it is time-consuming and labor intensive, making it impractical for large scale screening. The APEX technology is specifically designed to analyze previously reported sequence alterations and can detect hundreds of known mutations in parallel. However, it cannot detect novel mutations and has varying detection rates in different populations [6, 7]. Recently, next generation sequencing (NGS) has been used for diagnosing retinal diseases at the molecular level [20–26]. The NGS-based approach can detect both known and novel mutations among multiple genes in parallel.

Using this approach and the knowledge of previously reported RP and LCA genes (in RetNet at the time of system design, accessed 23th January 2014), we set up a custom designed targeted-NGS system to identify genetic defect in RP and LCA patients (Supplementary Table 1 in Supplementary Material available online at http://dx.doi.org/10.1155/2015/693468). In this study, we used our targeted-NGS approach in a set of Japanese fraternal (dizygotic) male twins with LCA.

## 2. Material and Methods

### 2.1. Patients and Clinical Evaluations

A set of Japanese male twins with LCA (family ID: LCA1H) were examined at the Department of Ophthalmology Hamamatsu University Hospital. The twins were dichorionic-diamniotic and showed differences in physical traits. Therefore, these findings led us to suspect that they were dizygotic. Complete history taken, pedigree analysis and ophthalmic examinations were performed (Table 1). This included visual acuity (Teller acuity cards) testing, slit-lamp biomicroscopy examination, and ophthalmoscopy examination. Cycloplegic refractive error was measured using an autorefractometer. Additionally, skin-electrode full-field ERGs were recorded using the protocol of the International Society for Clinical Electrophysiology of Vision [27]. Genomic DNA was extracted from peripheral lymphocytes of the twins and their parents using standard procedures. In addition, 200 unrelated Japanese individuals without RP were screened as controls to evaluate the incidence of mutations found in the twins.

### 2.2. Target Capture and Next Generation Sequencing

Library preparation for NGS was performed using the HaloPlex Target Enrichment kit 500 kb (Agilent Technologies, Santa Clara, CA, USA) according to the HaloPlex Target Enrichment System for Illumina Sequencing D.3. Using the Agilent SureDesign online tool (https://earray.chem.agilent.com/suredesign/index.htm), a custom target enrichment library was designed to capture the 74 genes known to be associated with RP or LCA, as reported in the RetNet at the time of system design (https://sph.uth.edu/retnet/; accessed 23th January 2014, Supplementary Tables  1 and 2). Probes were generated for 1182 regions to cover all exons and flanking intronic sequences (intronic sequence ±25 bp from exon boundaries) of these 74 genes. Amplicon libraries were prepared according to the manufacturer's instructions from genomic DNA of the twins. In brief, 225 ng of genomic DNA was used for restriction reactions, and hybridization was performed for 3 hours at 54°C. All DNA samples were individually indexed during hybridization step and library polymerase chain reaction (PCR) amplification was performed on the Thermal Cycler Dice TP600 (Takara Bio, Otus, Japan). Restriction digestion and concentration of amplicon libraries were quality controlled using a Bioanalyzer (Agilent Technologies, Santa Clara, CA, USA). Index sample concentrations were adjusted to 4 nM and combined to form the sample library. Finally, the dilute, denatured 6 pM sample library was loaded onto the Illumina (San Diego, CA, USA) MiSeq sequencer according to the manufacturer's instructions using 150 bp paired-end chemistry.

### 2.3. Next Generation Sequencing Data Analysis

All sequence and statistical analyses were performed using relevant programs in the Genomics Workbench software package, a commercially-available, stand-alone type program (version 7.5.1; CLC bio, Aarhus, Denmark). All sequence reads were initially trimmed to remove the parts of reads with low quality scores or short read length (<15 bp) using the Trim Sequences program with their default parameters. Trimmed sequence reads were mapped to the human reference genome (hg19) using the Map Reads to Reference program with the following settings: mismatch cost = 2, insertion cost = 3, deletion cost = 3, length fraction = 0.5, similarity fraction = 0.8, auto detected paired distance = “deselect,” and map to nonspecific reads = “ignore.” The Local Realignment program was then used to improve detection variation. Finally, the Fixed Ploidy Variant Detection and the InDels and Structural Variants programs were used to call sequence variations using the following filter parameters: minimum coverage = 50, minimum count = 10, minimum frequency (%) = 20, base quality filter = “active,” and relative read direction filter = “active.” Variation annotation was performed using the Amino Acid Changes, Annotate with Overlap Information, and Annotate from Known Variants programs with the single nucleotide polymorphism (SNP) database (dbSNP). To filter out possible nonpathogenic sequence alterations, all variations localized in intronic regions except within 5 bp from the coding exon flanking boundaries and in noncoding exonic regions were excluded. Common genetic variations (allele frequency > 0.34%) were identified using public SNP databases (NCBI dbSNP database [http://www.ncbi.nlm.nih.gov/projects/SNP/], 1000 Genomes database [http://www.1000genomes.org/], and Human Genetic Variation Browser database [HGVD, http://www.genome.med.kyoto-u.ac.jp/SnpDB/]) and 200 unrelated Japanese control subjects without RP. The benchmark frequency of 0.34% was determined based on the minor allele frequency of the most common Japanese arRP mutation EYS: p.S1653Kfs (rs527236065), as found in 729 control chromosomes in the HGVD. Because LCA is a rarer Mendelian disease than RP, if variation allele frequency was >0.34% in any of the public SNP databases, it would be treated as a possible nonpathogenic sequence alterations in this study (Supplementary Figure  1). Stringent filtering was used to exclude false positive variations. However, it should be noted that these steps might have also filtered out true pathogenic mutations. If a potential pathogenic mutation was not detected using the data pipeline, less stringent filtering criteria were applied (e.g., inclusion of variations localized in intronic regions within 25 bp from the exon flanking boundaries or in noncoding exonic regions). The 74 genes and gene accession numbers used for the mutation coordinates are shown in Supplementary Table 1. Statistical analyses of targeted region mapping density were performed using the Create Statistics for Target Regions program (Genomics Workbench, Table 2). For more information about the programs refer to the instruction manual available on the CLC website (http://clcsupport.com/clcgenomicsworkbench/current/index.php?manual=Introduction_CLC_Genomics_Workbench.html).

### 2.4. Genetic Variation Pathogenicity Assessment

A genetic variation was considered to be potentially pathogenic if it had been previously described as a pathogenic mutation with experimental evidence of the abnormal transcript, a nonsense mutation, a frameshift mutation, an in-frame mutation, or a mutation in the first 2 bases of canonical intron splice donor or acceptor sites. In particular, a missense mutation was described as potentially pathogenic when it fulfilled at least 2 of the following criteria: a conserved amino acid residue was affected; the* in silico* analysis predicted a pathogenic effect (see next section); and/or it was segregated with the disease phenotype within the family. Variations with a frequency > 0.34% were excluded from being classified as pathogenic, even when they were previously described as pathogenic mutations (see last section).

### 2.5. *In Silico* Analyses to Assess Missense or Splice Site Mutation Pathogenicity

The following 5 computational algorithms were used to evaluate missense mutation pathogenicity: SIFT (http://sift.jcvi.org/www/SIFT_seq_submit2.html), PolyPhen2 (http://genetics.bwh.harvard.edu/pph2/), PMut (http://mmb.pcb.ub.es/PMut/), SNAP (http://rostlab.org/services/snap/), and Align-GVGD (http://agvgd.iarc.fr/agvgd_input.php) [28]. The secondary structure of RetGC-1, the protein encoded by* GUCY2D*, was predicted using PSIPRED (v3.3) on the PSIPRED server (http://bioinf.cs.ucl.ac.uk/psipred/). Splice site mutations were analyzed using the Splice Site Prediction by Neural Network software (http://www.fruitfly.org/seq_tools/splice.html).

### 2.6. Sanger Sequencing Validation and Segregation Analyses

Potential pathogenic mutations detected by NGS were validated using standard protocol Sanger sequencing [29]. Sanger sequencing segregation analyses were performed on DNA from family members to investigate cosegregation of potentially pathogenic mutations. The following primer sets were used in this study: exon 10 in* GUCY2D* forward primer 5′-GAGCAAGGGAACCAAGCAG-3′ and reverse primer 5′-AGGAATTGTGTCTGGTGGATGT-3′ and exon 14 in* GUCY2D* forward primer 5′-AGACCGGCTGCTTACACAGAT-3′ and reverse primer 5′-GGAATAAATAAGGGACAGGAGGTC-3′.

## 3. Results

### 3.1. Clinical Findings

**Figure 1:**
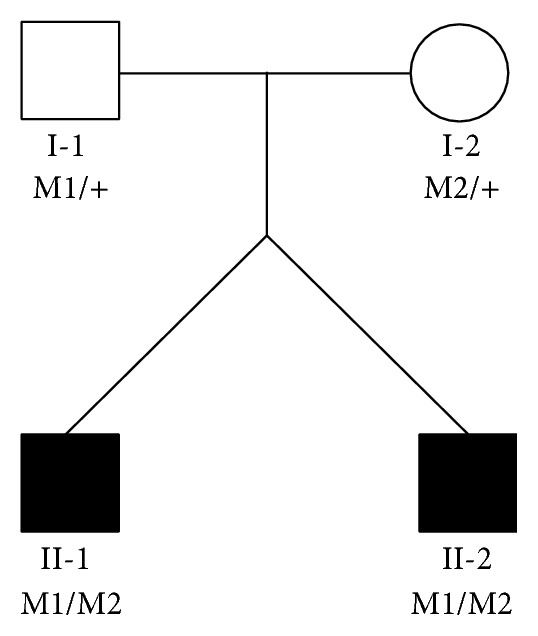
Pedigree of the LCA1H family. The nucleotide numbering reflects cDNA numbering with +1 corresponding to A of the ATG translation initiation codon in the reference sequence NM_000180, according to the nomenclature recommended by the Human Genome Variation Society (http://www.hgvs.org/mutnomen/). The initiation codon was designated as codon 1. The genotypes for the twins (II-1, II-2) and their parents (I-1, I-2) are shown with c.2113+2_2113+3insT denoted as M1 and c. 2714T>C (p.L905P) denoted as M2. Squares indicate males and circles indicate females. Filled symbols indicate individuals affected by Leber congenital amaurosis. M1/+ and M2/+ indicate heterozygous carriers. M1/M2 represents individuals presenting both mutations as compound heterozygous.

**Figure 2:**
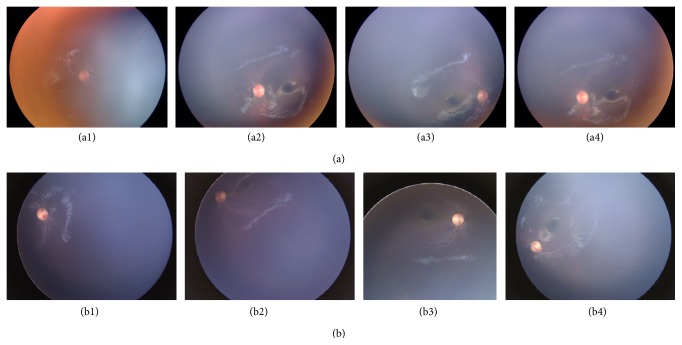
Fundoscopic photographs of twins affected by Leber congenital amaurosis. When the twins (II-1 and II-2) were 11 months old (II-1 right eye: (a1), II-1 left eye: (a2), II-2 right eye: (a3), and II-2 left eye: (a4)), they had mild retinal pigment epithelium changes around the optic disc and slight narrowing of the retinal vessels. Neither pigmentary changes nor macular degeneration were observed in either twin. When the twins were 29 months old (II-1 right eye: (b1), II-1 left eye: (b2), II-2 right eye: (b3), and II-2 left eye: (b4)), no further retinal changes had occurred.

A set of male twins in the LCA1H family were suspected to be affected by a recessively inherited form of LCA (Figure 1). The twins (II-1 and II-2) were born in the Hamamatsu area to parents who are not related to each other. Physical examination at 3 months of age revealed the absence of ocular pursuit in both twins, and they presented to our clinic for the first time at 11 months of age. At this time, the oculodigital sign, hyperopia, and nystagmus were present. Bilateral fundus examination of both twins showed only mild retinal pigment epithelium changes around the optic disc, slight retinal vessels narrowing, and no pigmentary changes. However, ERG recordings with a skin electrode had an extinguished pattern. The reexamination performed at 29 months of age revealed signs of severe photophobia in both twins. No significant retinal changes occurred during the follow-up period (Figure 2, Table 1) and no other clinical abnormalities were detected on physical examination, which included intracranial and neurological testing. With the exception of refractive error, there were no differences in the clinical presentations of the twins. Normal-sighted parents did not agree to receive the ophthalmic examinations.

### 3.2. Targeted Next Generation Sequencing Results

Each capture panel consisted of 445,968 bp that examined 1182 target regions of 74 genes (Supplementary Tables 1 and 2). Target region-enriched DNA was sequenced using NGS. The mean number of reads generated was 2,188,396.5, of which approximately 90% were mapped to targeted regions (Table 2). Targeted regions had an average of 269.1 ± 7.7-fold coverage among all samples. Additionally, an average of 95.5% of bases in the target region had a coverage of 20-fold and 91.6% had a coverage of 40-fold. These results indicate that sufficient coverage to identify variations was achieved. Individual data from each twin are shown in Table 2.

### 3.3. Potential Pathogenic Mutations in the LCA1H Twins

Twin 1 (II-1) and twin 2 (II-2) had 618 and 622 raw variations identified, respectively. They were initially found using automated variation detection compared to a reference sequence. After filtering out variations in intronic regions except within 5 bp from coding exon flanking boundaries and in noncoding exonic regions, 105 and 110 variations remained in twin 1 and twin 2, respectively. After excluding common (>0.34% allele frequency in any public SNP database) and synonymous variations, the same 2 rare variations remained in both twins. Consequently, compound heterozygous mutations of a novel splicing mutation c.2113+2_2113+3insT and a novel missense mutation c. 2714T>C (p.L905P) of* GUCY2D* were detected in both twins.

The* GUCY2D* encodes the photoreceptor membrane specific guanylate cyclase (RetGC-1), which catalyzes the conversion of guanosine triphosphate (GTP) to cyclic 3′,5′-guanosine monophosphate (cGMP) in the photoreceptor. The amino acid residue at L905, whose codon is affected by the mutations identified in the twins, is located in the catalytic domain (CD) of the corresponding RetGC-1 protein. The L905 residue was compared with those encoded by orthologous genes of several vertebrates (cow, dog, rat, mouse, and zebrafish) and the fruit fly and was found to be highly conserved across species (Table 3). Additionally, the p.L905P mutation was predicted to be pathogenic by 5 different computational prediction programs (SIFT, Polyphen2, SNAP, PMut, and Align-GVGD; Table 3). The p.L905P mutation was analyzed by the PSIPRED program to determine its effect on RetGC-1 secondary structure. This predicted cleavage of the alpha-helix structure from 19 contiguous amino acids (898-PIEVVDLLNDLYTLFDAII-916) to 5 (899-IEVVD-903) and 10 (907-DLYTLFDAII-916) contiguous amino acids. This suggested that the p.L905P mutation is deleterious to the helical segment and that it affects normal protein structure. On the other hand, splicing mutation c.2113+2_2113+3insT was located in the intron 10 donor site of* GUCY2D*. To predict the degree to which the nucleotide substitution in splice sites affect splicing, we performed* in silico* analyses using splice site prediction (Splice Site Prediction by Neural Network). The splice donor site score of the normal allele was 0.86, suggesting a high enough ability for splicing (score range of 0-1 with a larger score indicating a greater ability), but the mutant allele was not recognized as a splicing donor site. Therefore, our results suggest that the c.2113+2_2113+3insT mutation causes intron 10 to remain in the transcript and be translated. This would produce a stop codon 2 amino acids downstream, possibly resulting in a nonsense-mediated mRNA decay (NMD) [30].

Sanger sequencing results confirmed 2 novel potentially pathogenic mutations. Additionally, they were found to cosegregate with disease phenotype because the unaffected father (I-1) and unaffected mother (I-2) were heterozygous for c.2113+2_2113+3insT and p.L905P, respectively (Figure 1, Supplementary Figure  2). Neither of these potentially pathogenic mutations was found in our Japanese controls or in any of the public SNP databases (Table 3).

## 4. Discussion

In this study, we described the genetic and clinical features of a set of Japanese male twins with LCA. Two novel potentially pathogenic mutations in* GUCY2D* were detected in both twins using targeted-NGS. A number of potential pathogenic mutations have already been identified in LCA-affected families of various ethnic backgrounds, and several have described mutations in Japanese LCA families [7, 31–33]. However, to the best of our knowledge, this is the first study to report on* GUCY2D* mutations in a Japanese LCA family.

The* GUCY2D* was the first gene associated with LCA pathogenesis [34] and accounts for approximately 12% of all LCA cases [5]. The RetGC-1 protein is encoded by* GUCY2D* and has the following 5 distinct domains: extracellular domain (ECD), transmembrane domain, intracellular kinase homology domain (KHD), dimerization domain, and catalytic domain (CD). The majority of mutations detected in LCA patients are located in the ECD, KHD, and CD of the RetGC-1 protein [3, 6, 8].

In this study, compound heterozygous* GUCY2D* mutations of c.2113+2_2113+3insT and p.L905P were detected in a set of Japanese twins with LCA. The mutations were cosegregated with the LCA phenotype and neither was present in our control group of normal Japanese subjects or found in any public SNP database. The c.2113+2_2113+3insT mutation was predicted to produce a transcript that may be an NMD target, resulting in no protein product. The p.L905P mutation was predicted to be pathogenic by* in silico* analysis (Table 3). The amino acid residue at L905 was located in the RetGC-1 CD and was found to be highly conserved across species (Table 3). A previous study reported that the majority of RetGC-1 missense mutations located in the CD result in complete elimination of cyclase activity [35]. Therefore, we believe that the 2 novel mutations identified in this set of twins are pathogenic.

The phenotypic features of the twins examined here were similar to those previously reported in patients with* GUCY2D* mutations. Symptoms included visual loss, nystagmus, unrecordable ERG, photophobia, and hyperopia since early childhood [3, 36]. Unfortunately, the twins were not cooperative during fundus examinations and had severe nystagmus, both of which prevented detection of subtle macular abnormalities. The natural course of the visual function corresponding to the specific LCA gene defect has reported that patients with* AIPL1*,* RPGRIP1*, and* RDH12* mutations exhibit a degenerative type of disease and have a steady decline in vision, patients with* CRB1* and* RPE65* mutations exhibit a transient improvement in visual function but have an eventual deterioration in vision, and patients with* GUCY2D* and* CEP290* mutations have a stagnant disease with relatively stable, albeit poor, and visual function [34, 37, 38]. Thus, identifying specific* GUCY2D* mutations in a patient through precise genetic counseling can lead to a more accurate prognosis of vision and assessment of disease state. We assume that visual function in the twins will remain stable in later years.

Gene therapy seems promising for treating LCA patients. An accurate molecular diagnosis is a prerequisite for selecting patients for clinical trials and is essential for determining potential therapeutic interventions, particularly for gene therapy. Additionally, different retinal diseases have many overlapping clinical features, which can make it difficult to accurately diagnose a patient. However, this study suggests that a targeted-NGS approach could be useful for potential gene-specific therapeutic interventions.

## 5. Conclusions

We used a targeted-NGS approach to identify genetic defects in a set of Japanese male twins with LCA. Consequently, compound heterozygous mutations were identified in both twins and included a novel splicing mutation (c.2113+2_2113+3insT) and a novel missense mutation (p.L905P) in* GUCY2D*. Both twins had similar clinical features, which included visual loss, poor light reflex, nystagmus, and an extinguished ERG response, suggesting that the twins had LCA. We believe the LCA was possibly caused by the 2 identified, potentially pathogenic, mutations in* GUCY2D*. This study is the first one reporting LCA in Japanese patients with* GUCY2D* mutations.

## Supplementary Material

Supplementary Table 1. List of the 74 genes captured in the present study.
A custom target enrichment library was designed to capture these 74 genes in this list known to be associated with RP or LCA, as reported in the RetNet at the time of system design (; accessed 23th January 2014 ).
Supplementary Table 2. List of all probes used to enrich for the target genes.
Using the Agilent SureDesign online tool (https://earray.chem.agilent.com/suredesign/index.htm), probes were generated for 1182 regions in this list to cover all exons and flanking intronic sequences (intronic sequence ± 25 bp from exon boundaries) of the 74 genes.

## Figures and Tables

**Table 1:** Clinical characteristics of male twins affected by Leber congenital amaurosis.

Family ID	Patient	Age at onset (months)	Age at first visit (months)	Current age (months)	Follow-up duration (months)	Gender	Origin	Inheritance pattern	Primary symptom	Night blindness	Photophobia	Nystagmus	Hyperopia	Refraction (diopters)		ERG		Fundus abnormalities
														Right eye	Left eye	Right eye	Left eye	
LCA1H	Twin 1 (II-1)	3	11	29	18	Male	Hamamatsu, Japan	ar	No ocular pursuit	−	+	+	+	+3.00	+3.25	NR	NR	Mild RPE changes around disc. Slight retinal vessels narrowing.
	Twin 2 (II-2)	3	11	29	18	Male	Hamamatsu, Japan	ar	No ocular pursuit	−	+	+	+	+6.00	+5.00	NR	NR	Mild RPE changes around disc. Slight retinal vessels narrowing.

Age at onset was based on medical history; age at first visit was based on medical records.

All clinical data were obtained from recent examinations.

ar: autosomal recessive; ERG: electroretinogram; NR: nonrecordable; RPE: retinal pigment epithelium.

**Table 2:** Quality of the targeted-next generation sequencing in this study.

Sample	Number of target regions	Total length of target regions (bp)	Total mapped reads	Mapped reads in targeted region	Specificity (%)	Average coverage	Target covered with at least 1x (%)	Target covered with at least 20x (%)	Target covered with at least 40x (%)
Twin 1 (II-1)	1182	445,968	2,136,143	1,850,847	89.93	263.6	98.56	95.51	91.68
Twin 2 (II-2)	1182	445,968	2,240,650	1,926,085	89.36	274.5	98.58	95.53	91.47

**Table 3:** Characteristics of potential pathogenic mutations identified in this study.

	Nucleotide change	Predicted effect	Location in gene	Domain^a^	Conservation across species^b^	Control allele frequency	SNP ID	Computational prediction^c^				
								SIFT	PolyPhen2	PMut	SNAP	Align-GVGD
Splicing	c.2113+2_2113+3insT		Intron 10		Not applicable	0/400	Not present					
Missense	c.2714T>C	p.L905P	Exon 14	CD	L/L/L/L/L/L/L	0/400	Not present	Damaging (score 0.00)	Probably damaging	Pathological	Nonneutral	C65

^a^indicates RetGC-1 (protein encoded by *GUCY2D*) domain; CD = catalytic domain.

^b^denotes human/cow/dog/rat/mouse/zebrafish/fruit fly *GUCY2D* orthologs (sequences selected from the DDBJ/EMBL/GenBank database).

Accession numbers were NM_000180 (human), NM_174548 (cow), NM_001003207 (dog), NM_024380 (rat), NM_008192 (mouse), NM_131866 (zebrafish), and NM_001202237 (fruit fly). L indicates leucine.

^c^All models used to evaluate the missense mutation suggested that the mutation is pathogenic.

The Align-GVGD analysis results are graded from C0 to C65, where C0 indicates a benign mutation and C65 indicates that the mutation is most likely pathogenic.
