# Supplementary material for: Novel GUCY2D Gene Mutations in Japanese Male Twins with Leber Congenital Amaurosis
Source: J Ophthalmol. 2015 May 13;2015:693468. doi: 10.1155/2015/693468 (PMC4444599; doi:10.1155/2015/693468)

**Novel *GUCY2D* gene mutations in Japanese male twins with Leber congenital amaurosis**

Supplementary Information

Supplementary Figure 1. Data analysis pipeline.

Schematic of data processing methods used to filter and identify potential pathogenic mutations in this study. Stringent filtering and prioritization were used to exclude false positive variations, but may have also excluded true pathogenic mutations.

Supplementary Figure 2. Electropherograms of potential pathogenic *GUCY2D* mutations.

Partial *GUCY2D* sequence is shown for twin 1 (II-1: A-1 and A-2), twin 2 (II-2: B-1 and B-2), the father (I-1: C-1 and C-2), and the mother (I-2: D-1 and D-2). Deduced amino acids are indicated under the sequence trace and mutation location is indicated by an arrow. The c.2113+2_2113+3insT was located in intron 10 and the c. 2714T>C (p.L905P) mutation was located in exon 14. The unaffected father and mother were heterozygous for c.2113+2_2113+3insT and p.L905P, respectively.

Supplementary Table 1. List of the 74 genes captured in the present study.

Supplementary Table 2. List of all probes used to enrich for the target genes.

Supplementary Figure 1





Supplementary Figure 2


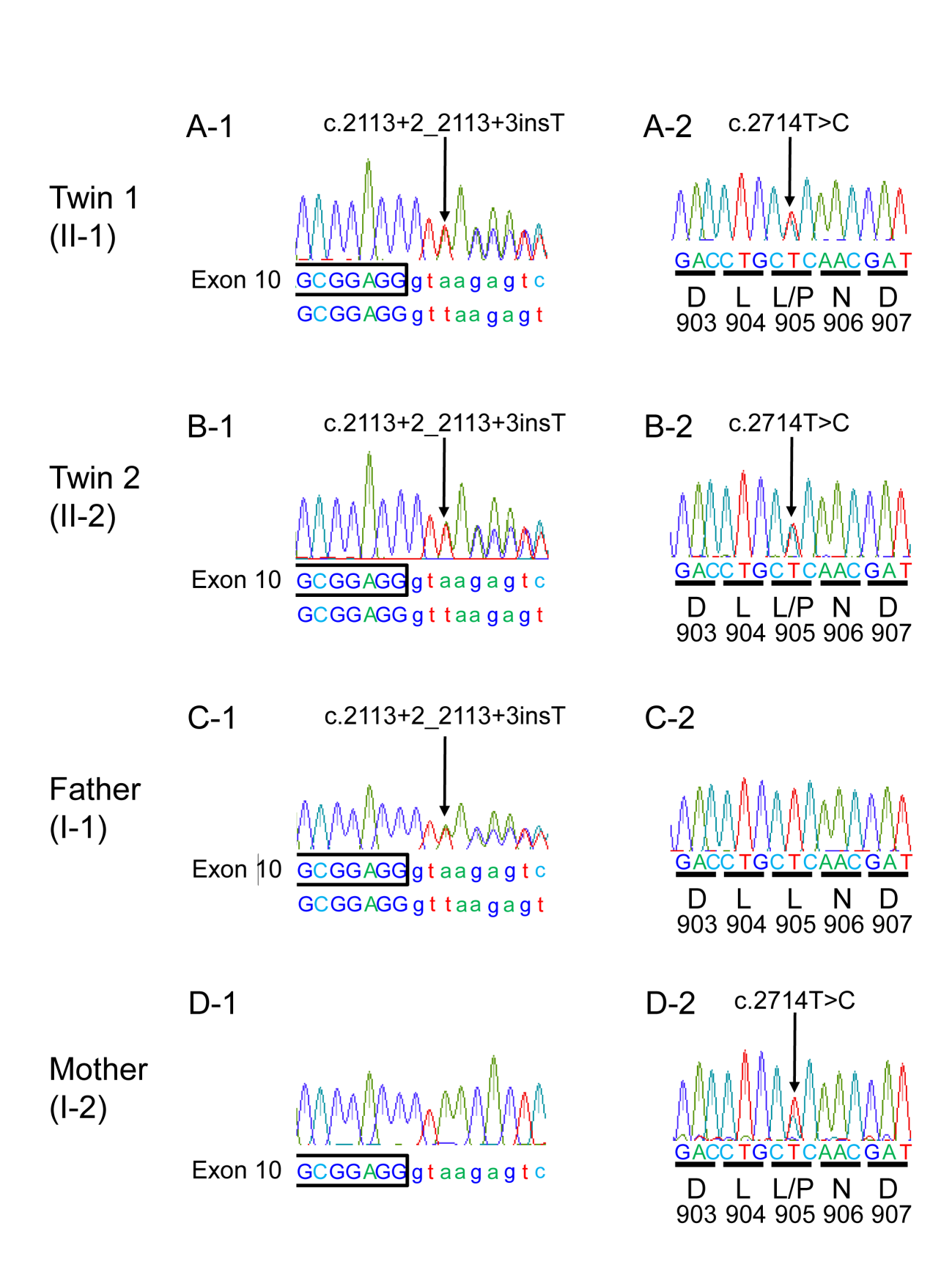

Supplement: Supplementary file 1 — Supplementary Table 1. List of the 74 genes captured in the present study. A custom target enrichment library was designed to capture these 74 genes in this list known to be associated with RP or LCA, as reported in the RetNet at the time of system design (; accessed 23th January 2014 ). Supplementary Table 2. List of all probes used to enrich for the target genes. Using the Agilent SureDesign online tool (https://earray.chem.agilent.com/suredesign/index.htm), probes were generated for 1182 regions in this list to cover all exons and flanking intronic sequences (intronic sequence ± 25 bp from exon boundaries) of the 74 genes. [file 693468.f1.docx]
